# Aspiration Therapy As a Tool to Treat Obesity: 1- to 4-Year Results in a 201-Patient Multi-Center Post-Market European Registry Study

**DOI:** 10.1007/s11695-017-3096-5

**Published:** 2018-02-01

**Authors:** Max Nyström, Evzen Machytka, Erik Norén, Pier Alberto Testoni, Ignace Janssen, Jesus Turró Homedes, Jorge Carlos Espinos Perez, Roman Turro Arau

**Affiliations:** 1 Department of Surgery, Blekinge County Hospital, Lasarettsvägen, 371 85 Karlskrona, Sweden; 2 University Hospital Ostrava, Pionyru 690, 250 81 Nehvizdy, Czech Republic; 3 Department of Gastroenterology, Ospedale San Raffaele, Via Olgctnna, 60, 20132 Milan, Italy; 4 Dutch Obesity Clinics, Amersfoortseweg 43, 3712 BA Huis ter Heide, The Netherlands; 5 Endoscopia Digestiv, Centro Medico Teknon, Carrer Vilana, 12, 08022 Barcelona, Spain

**Keywords:** Aspiration therapy endoscopic weight loss

## Abstract

**Purpose:**

The objective of this post-market study was to evaluate long-term safety and efficacy of aspiration therapy (AT) in a clinical setting in five European clinics.

**Materials and Methods:**

The AspireAssist® System (Aspire Bariatrics, Inc. King of Prussia, PA) is an endoscopic weight loss therapy utilizing a customized percutaneous endoscopic gastrostomy tube and an external device to aspirate approximately 30% of ingested calories after a meal, in conjunction with lifestyle counseling. A total of 201 participants, with body mass index (BMI) of 35.0–70.0 kg/m^2^, were enrolled in this study from June 2012 to December 2016. Mean baseline BMI was 43.6 ± 7.2 kg/m^2^.

**Results:**

Mean percent total weight loss at 1, 2, 3, and 4 years, respectively, was 18.2% ± 9.4% (*n*/*N* = 155/173), 19.8% ± 11.3% (*n*/*N* = 82/114), 21.3% ± 9.6% (*n*/*N* = 24/43), and 19.2% ± 13.1% (*n*/*N* = 12/30), where *n* is the number of measured participants and *N* is the number of participants in the absence of withdrawals or lost to follow-up. Clinically significant reductions in glycated hemoglobin (HbA1C), triglycerides, and blood pressure were observed. For participants with diabetes, HbA1C decreased by 1% (*P* < 0.0001) from 7.8% at baseline to 6.8% at 1 year. The only serious complications were buried bumpers, experienced by seven participants and resolved by removal/replacement of the A-Tube, and a single case of peritonitis, resolved with a 2-day course of intravenous antibiotics.

**Conclusion:**

This study establishes that aspiration therapy is a safe, effective, and durable weight loss therapy in people with classes II and III obesity in a clinical setting.

**Trial Registration:**

ISRCTN 49958132

**Electronic supplementary material:**

The online version of this article (10.1007/s11695-017-3096-5) contains supplementary material, which is available to authorized users.

## Introduction

Obesity is arguably the most significant global public health issue due to its high prevalence and adverse effects on health, quality of life, and health care costs [1]. Although the obesity rate has risen significantly in most countries over the past 15 years [2], class II (body mass index [BMI] 35.0–39.9 kg/m^2^) and class III obesity (BMI ≥ 40.0 kg/m^2^) rates have risen even more sharply across many countries [3–7]. This trending is alarming in that there is a significantly elevated risk of premature death primarily due to heart disease, cancer, and diabetes with increasing BMI [8].

Conservative weight loss therapies (lifestyle modification and pharmacotherapy) are rarely successful at maintaining weight loss over the long-term [9]. Bariatric surgery provides significant and durable weight loss, as well as a reduction in obesity-related comorbidities, but at an elevated risk of both short-term and long-term complications. Less than 2% of the patients with class II or class III obesity undergo either conservative therapies or bariatric surgery (“conventional therapies”) in any 1 year [10, 11]. As a result, there have been significant efforts over the past decade to develop endoscopic bariatric therapies for patients with class II or class III obesity who either do not desire, or do not qualify for, bariatric surgery.

Aspiration therapy (AT) is an endoscopic weight loss therapy utilizing a novel device, the AspireAssist®. The device is easily removable in a simple endoscopic procedure. The AspireAssist system is commercially available in Europe and the USA, and more recently in Australia, Canada, and Israel. It is labeled for long-term use for people with BMIs of 35–65 kg/m^2^ in Europe and Australia and for people with BMIs of 35–55 kg/m^2^ in the USA [12] and Canada. The system allows partial drainage of gastric contents after a meal and consists of a percutaneous endoscopic gastrostomy tube (A-Tube™) and an external detachable device to facilitate drainage. In a previous study, it was shown that a maximum of about 30% of the calories consumed in a meal could be aspirated with this system [13]. Lifestyle counseling/cognitive behavior therapy (CPT) was provided conjunctively with this therapy. This device has been the subject of several prior studies, including 2-year results in an 18-subject, single-center randomized controlled trial (RCT) in the USA [13]; 1-year results in a 171-subject, multi-center RCT in the USA (“PATHWAY Study”) [14]; and 2-year results in a 25-subject single-center observational study in Sweden [15]. Significant weight loss was achieved in these studies, and the complications reported were generally minor and similar in type, severity, and frequency to those reported in the literature with PEG tubes [16–19]. There was no indication of any clinically significant metabolic abnormality or deterioration in eating behaviors for any AT participant in these three studies.

The present report describes a multi-year post-market registry study designed to evaluate the efficacy, durability, and safety of the AspireAssist for weight management in persons with class II and class III obesity in a “real-world” clinical setting.

## Methods

A post-market observational registry study was performed at five centers in Europe. A total of 201 participants were enrolled from June 2012 to December 2016. All patients who elected to undergo aspiration therapy between June 2012 and December 2016 in the above clinics were included in this registry/observational study. Participants were recruited either on a first-come-first-serve basis via advertisements in local media or by presentation to the clinic in search of a minimally invasive weight loss procedure.

Key inclusion criteria were age 18 years old or older, a BMI greater than 35.0 kg/m^2^, and prior failed attempts utilizing conservative weight loss methods. Key exclusion criteria were known malignancy, serious cardiovascular disease, chronic liver or kidney disease, prior abdominal surgery that would increase the risk of endoscopic A-Tube placement, a history of major depressive or other severe psychiatric disorders, including any eating disorder, or an intellectual disability. Participants were allowed to continue the therapy for as long as the participant desired.

The protocol was substantially uniform across all the sites, with minor differences to accommodate each clinic’s standard weight loss/bariatric/bariatric endoscopy program and improvements in the clinic’s AT program, as knowledge of the therapy accumulated (Suppl. Table S1). Prior to initiation of AT, the A-Tube, a customized gastrostomy tube with a 15-cm fenestrated intra-gastric portion (to facilitate gastric drainage), was placed in a near identical endoscopic procedure to a percutaneous endoscopic gastrostomy (PEG) tube placement, utilizing the pull-technique [20, 21]. Prophylactic antibiotics were administered. A-Tube placement was performed on an outpatient basis, using conscious sedation in all participants, except for eight participants with BMI > 60 who were either intubated (*n* = 2) or admitted for an overnight stay (*n* = 6). When the gastrostomy matured, at approximately 10–14 days following A-Tube placement, the external end of the A-Tube was cut to within 1 cm of the abdominal wall and attached to a Skin-Port, a low-profile “button” valve. Patients were then instructed on how to use the device, to aspirate 20–30 min after each of the three daily meals, and to chew very thoroughly to facilitate drainage and to avoid A-Tube blockage.

During the first year, participants returned to the clinic approximately once per month for medical checkups (weight and vital signs, inspection of the stoma site, review of blood work, and any issues) and lifestyle/cognitive behavior therapy. Participants returned more frequently if medically warranted. In addition, some clinics offered group support meetings. After the first year, participants returned to the clinic approximately once every 3–6 months for checkups. In some cases, because of a participant’s work schedule or an excessive travel distance, follow-up was done electronically/telephonically, and in other cases, the participant’s care was managed by his or her general practitioner (GP). Lifestyle/cognitive behavior therapy varied from clinic to clinic. In some cases, it was taught by a certified cognitive behavior therapist or psychologist and, in other cases, by a dietician. The topics included (i) the fundamentals of aspiration therapy (chewing thoroughly, aspirating regularly, meal planning, regular structured meals), (ii) nutritional education (portion sizes, food labels, wise food choices), (iii) a behavioral program (mindful eating, self-monitoring, goal-setting, problem-solving, dealing with high-risk situations, and stress management habits), and (iv) a physical program (importance of physical activity and strategies to increase activity).

**Fig. 1:**
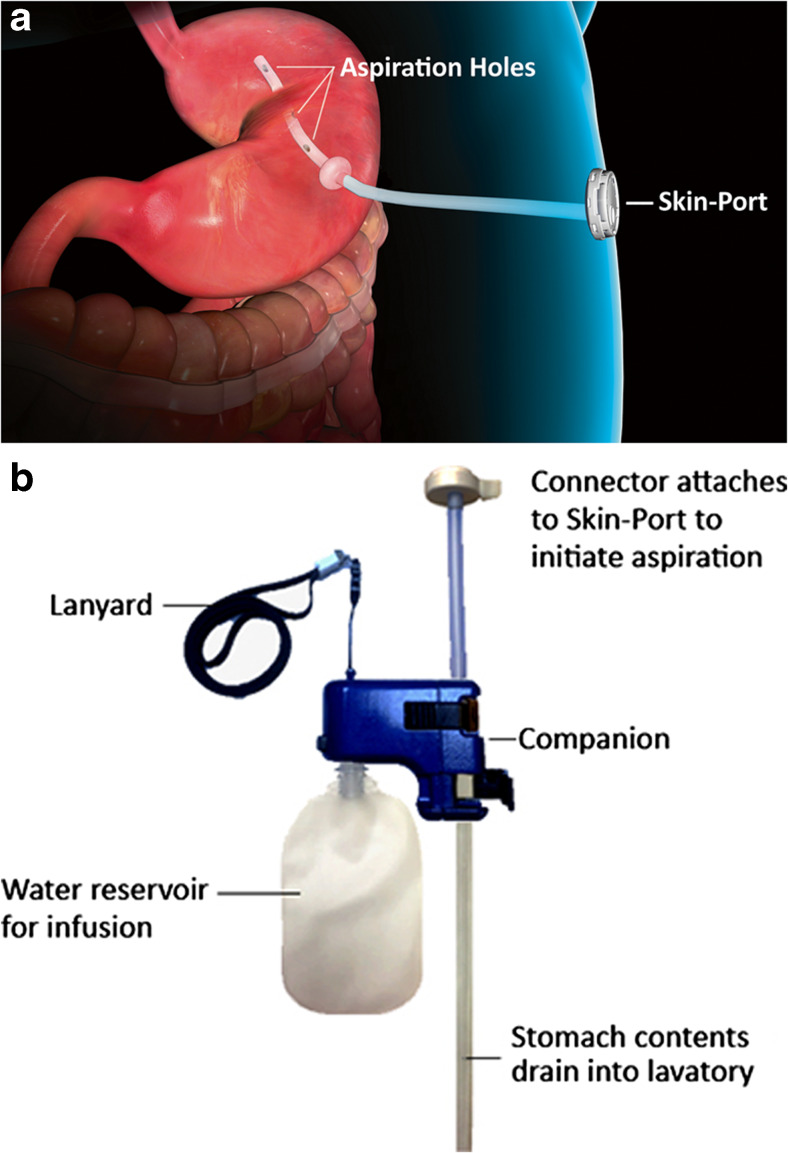
AspireAssist A-Tube with Skin-Port and external device**.** Individual components of the AspireAssist device are labeled

The components of the AspireAssist system include the A-Tube and Skin-Port (Fig. 1a) and a detachable handheld device (Fig. 1b), which provides two modes of operation: drainage and lavage. This system does not contain a pump, but rather relies upon the slight positive (relative to ambient) pressure of the stomach, and gravity, to allow drainage. About 20–30 min after a meal, in the privacy of a lavatory, the patient connects the device (Fig. 1b). This drain/irrigation process is repeated several times until the aspirate appears clear. The entire aspiration process usually takes 5–10 min to perform if the patient chews thoroughly. If a patient fails to chew thoroughly, the aspiration process may take longer and be less efficient (e.g., less than 30% of caloric content of the meal is removed) as the tube will repeatedly clog after irrigation when it resides among large food particles.

## Results

**Fig. 2:**
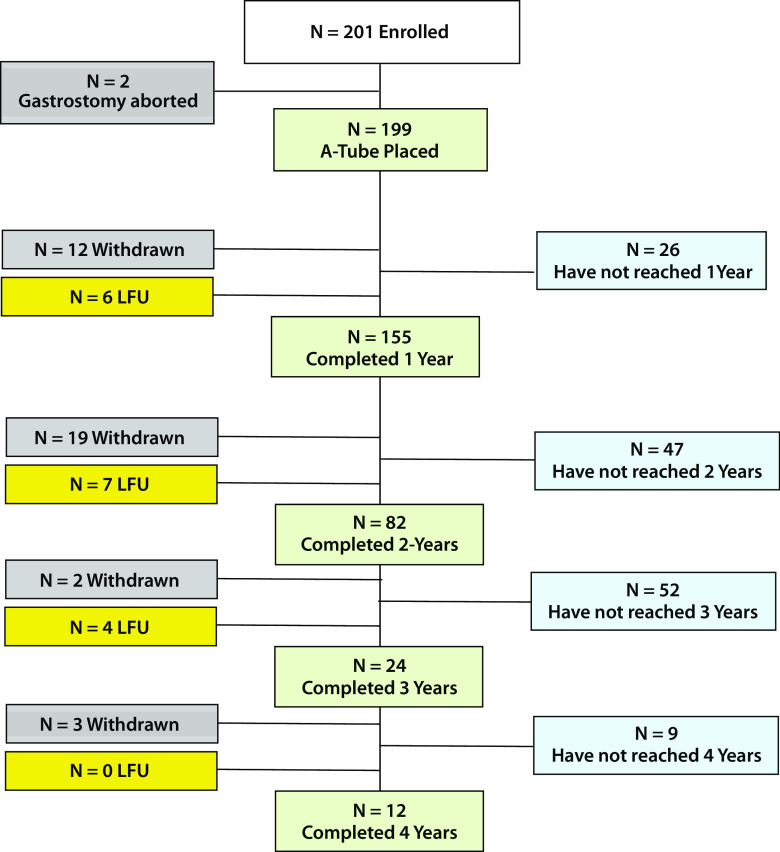
Participant disposition

A total of 201 participants (151F/50M) were enrolled from June 2012 to December 2016. All patients who elected to undergo AT between June 2012 and December 2016 in the five clinics represented by the investigators were included in this registry/observational study. Mean age at the time of starting the therapy was 46.1 ± 10.9 years (range 19–74 years). Baseline BMI was 43.6 ± 7.4 kg/m^2^ (range 35–74 kg/m^2^). As of March 31, 2017, 173, 114, 42, and 30 participants started AT therapy at least 1, 2, 3, and 4 years ago, respectively. Because of withdrawals or lost to follow-up, weight measurements were taken for only 155, 82, 24, and 12 participants at years 1, 2, 3, and 4, respectively (see the participant disposition chart, Fig. 2). In this study, lost to follow-up participants are participants who were overdue for their most recent annual visit as of March 31, 2017, typically < 3 months overdue. From our experience, most lost to follow-up participants will return to the clinic to continue AT and obtain a new connector. Participants which discontinue AT and request A-Tube removal are considered withdrawn.

### Weight Loss

For the modified intent to treat (mITT) population (excluding two participants with aborted gastrostomies and three participants who withdrew from the study prior to the gastrostomy), using a last observation carry-forward (LOCF) imputation for the18 participants who either withdrew from the therapy or were lost to follow-up, mean total weight loss (%TWL) (*n* = 173) at 1 year was 17.1% ± 9.6%. For the per-protocol population (*n* = 155), mean %TWL at 1 year was 18.2% ± 9.4%. Table 1 summarizes 1-year weight loss data and includes percent excess weight loss (%EWL) and absolute weight loss (AWL [kg]). At 1 year, of the mITT and per-protocol populations, 75 and 81%, respectively, achieved a minimum of 10% TWL, and 74 and 77%, respectively, achieved a minimum of 25% EWL.

**Table 1:** Mean 1-year weight loss results, with standard deviation and 95% confidence intervals, for modified intent to treat (mITT) and per-protocol populations. %total weight loss [%TWL], % excess weight loss [%EWL], and absolute weight loss [AWL] in kilograms

	*N*	%TWL	%EWL	AWL (kg)
mITT	173	17.1% ± 9.6% 95% CI 15.6%, 18.5%	43.6% ± 26.4% 95% CI 39.6%, 47.6%	21.3 ± 13.4 95% CI 193, 23.3
Per protocol	155	18.2% ± 9.4% 95% CI 16.7%, 19.7%	46.3% ± 26.3% 95% CI 42.1%, 50.5%	22.7 ± 133 95% CI 20.6, 24.8

*N* the number of participants measured

### Weight Loss Durability

Mean %EWL, %TWL, and AWL for participants who completed 1, 2, 3, and 4 years of therapy are presented in Table 2. So, as to not conflate durability of weight loss results with weight loss associated with different participant populations, we also examined weight loss for each completed year for all participants who completed 1, 2, 3, and 4 years of therapy (Suppl. Table. S1). As can be seen from the table and figure, first-year weight loss is maintained through at least the end of the fourth year. Weight loss (%TWL) in older participants (≥ 55 years) versus that of younger participants (< 55 years) (Suppl. Figure S2) and in participants with higher BMIs (≥ 50 kg/m^2^) versus lower BMIs (< 50 kg/m^2^) (Suppl. Figure S3) was investigated. Older and higher BMI participants trended towards greater weight loss (%TWL) than their younger and lower BMI counterparts, respectively, but the difference was not statistically significant.

**Table 2:** Mean weight loss, with standard deviation and 95% confidence intervals (CI), for year 1 through year 4 completers. percent total weight loss (%TWL), mean percent excess weight loss (%EWL), and mean absolute weight loss for years 1–4

	*n*/*N*	%TWL	%EWL	AWL (kg)
Year 1	155/173	18.2% ± 9.4% 95% CI 16.7%, 19.7%	46.3% ± 26.3% 95% CI 42.1%, 50.5%	22.7 ± 13.3 95% CI 20.6, 24.8
Year 2	82/114	19.8% ± 11.3% 95% CI 17.3%, 22.3%	48.2% ± 28.2% 95% CI 42.0%, 54.4%	25.8 ± 17.3 95% CI 22.0, 29.6
Year 3	24/42	21.3% ± 9.6% 95% CI 17.2%, 25.4%	50.3% ± 26.2% 95% CI 39.2%, 61.4%	29.1 ± 16.2 95% CI 22.3, 35.9
Year 4	12/30	19.2% ± 13.1% 95% CI 10.9%, 27.5%	47.9% ± 36.2% 95% CI 24.9, 70.9	25.1 ± 19.1 95% CI 13.0, 37.2

*n* is the size of the measured protocol population. *N* is the size of the available population, if no drop-outs or lost to follow-up

### Cardiometabolic Improvement

Although all sites monitored electrolytes, only two study sites monitored cardiometabolic status (blood pressure, lipids, glucose, glycated hemoglobin [HbA1C]); hence cardiometabolic data is only available from two sites.

With the exception of total cholesterol, clinically significant improvement in 1-year cardiometabolic data from baseline was observed. [HbA1C dropped 0.39% (*p* < 0.0001) from a 5.9% baseline; fasting glucose dropped 7.9 mg/dl (*p* < 0.01) from a 110 mg/dl baseline; systolic blood pressure dropped 12.1 mmHg (*p* < 0.0001) from a 141 mmHg baseline; diastolic blood pressure dropped 6.0 mmHg (*p* < 0.001) from a baseline of 88 mmHg; triglycerides dropped 25.5 mg/dl (*p* < 0.001) from a baseline of 133 mg/dl, and total cholesterol increased 4.3 mg/dl (*p* < 0.01) from a baseline of 186 mg/dl] (Table 3). For participants with diabetes, a 1.0% (*p* < 0.0001) mean reduction in 1-year HbA1C from 7.8% at baseline to 6.8% was observed.

**Table 3:** Mean cardiometabolic parameters at baseline and at 52 weeks and change at 1 year over baseline. glycated hemoglobin (HbA1C), glucose, blood pressure (BP), total cholesterol (CHO), and triglycerides, and glycated hemoglobin (HbA1C) for participants with diabetes only. Also shown is the change at 1-year from baseline, the 95% confidence intervals for the change from baseline, and the *P* value

	*N*	Baseline	1 year	Change	95% CI	*P*
HbA1C (%)	57	5.9 ± 1.3	5.5 ± 1.0	− 0.39 ± 0.44	(− 0.51, − 0.27)	< 0.0001
Glucose (mg/dl)	58	109.7 ± 42.4	101.8 + 38.2	− 7.9 + 19.0	(− 12.9, − 2.9)	< 0.01
Systolic BP (mm, Hg)	63	141.3 ± 16.9	129.2 ± 18.8	− 12.1 ± 19.3	(− 16.9, − 7.2)	< 0.0001
Diastolic BP (mg, Hg)	63	88.4 ± 11.5	82.4 ± 11.0	− 6.0 ± 14.0	(− 9.5, − 2.5)	0.001
Total CHO (mg/dl)	56	184.9 ± 45.8	198.8 ± 36.6	13.9 ± 36.5	(4.1, 23.7)	< 0.01
Triglycerides (mg/dl)	53	132.6 ± 66.9	107.1 ± 52.5	− 25.5 ± 49.1	(− 39.0, − 12.0)	< 0.001
Participants with diabetes						
HbA1C (%)	13	7.8 ± 1.6	6.8 ± 1.3	− 1.0 ± 0.5	(− 1.3, − 0.7)	< 0.0001

### Complications

Table 4 lists all complications reported through March 31, 2017. In the periprocedural period (≤ 7 days post-gastrostomy), there was one serious complication, a case of peritonitis (without abscess), resolved with a 2-day course of intravenous antibiotics. Additionally, eight participants were hospitalized for benign pneumoperitoneum (likely, the result of insufflation) for observation and analgesic administration. In the post-procedural period (> 7 days post-gastrostomy), seven participants experienced a serious complication: all buried bumpers (two participants with two events each, seven patents with one event). The buried bumpers were treated by removal of the A-Tube, temporary replacement with a 20-French PEG tube, and subsequent replacement with an A-Tube. Although buried bumpers are a known complication of PEG tubes and are understood to be caused by excessive tension on the internal bumper against the gastric wall, it is not known what caused the excessive tension. The buried bumpers all occurred in the same clinic. The only complications that were not known complications of PEG tubes were three events (4% of all complications) of A-Tube rotation (e.g., where the internal end rotated from the fundus towards the pylorus) that occurred in two participants and treated by replacement of the A-Tube. Except for the complications discussed previously (peritonitis, buried bumpers, and A-Tube rotation), all other complications resolved spontaneously or with conservative therapies (oral analgesics, topical administrations, or oral antibiotics).

**Table 4:** Complications occurring in the periprocedural period (≤ 7 days from gastrostomy) and post-procedural period (> 7 days from gastrostomy)

Periprocedural complications	Number of events	Number of participants	% of participants
Pain	28	28	14.2
Possible/actual wound infection	12	12	6.1
Benign pneumoperitoneum	9	9	4.6
Vomiting/diarrhea	3	3	1.5
Peritonitis	1	1	0.5
Total	53	53	26.9
Post procedural complications	Number of events	Number of participants	Events per patient-year
Gastric leakage	12	10	0.040
Stomal irritation/granulation tissue	10	10	0.033
Infection/possible infection	9	9	0.030
Buried bumper	8	6	0.027
A-Tube rotation	3	2	0.010
Total	42	37	0.139

### Persistent Fistulas Post A-Tube Removal

Of the 47 A-Tubes that have been removed, there were four incidents of persistent fistulas (e.g., the fistula not spontaneously closing) after A-Tube removal, one each occurring in the 13th, 25th, 34th, and 42nd month post-gastrostomy. Two were treated with argon plasma coagulation (APC) and proton pump inhibitors (PPIs) and closed successfully on the first re-attempt; the third and fourth did not respond to the APC/PPI regimen but closed successfully on the second re-attempt, utilizing endoscopic clips. As there were more A-Tubes removed in the first and second years (35 total) versus the third and fourth year (12 total), the data suggests that the rate of persistent fistulas increases significantly after 2 years post-gastrostomy (Suppl. Table S3).

### A-Tube Replacement

Seven (7) A-Tubes reached, or were close to reaching, the end of their useful lives, and required replacement. Applying a Kaplan-Meier survival analysis on the A-Tube failure data, about 60% of participants at 4 years would have their original A-Tube (Suppl. Figure S2). We do not list A-Tube replacement in this report as a complication unless it occurred within 12 months of placement because it is understood that the A-Tubes have a finite lifetime.

The failed A-Tubes (Suppl. Figure S3) typically either were pitted or developed a bulge in the ~ 1-cm-length extra-fistular region between the skin and the Skin-Port. In three cases, the A-Tube developed either a leak or a tear in this same region, and in the other four cases, they were removed prior to a failure. The failure analysis by the manufacturer, Aspire Bariatrics, was a fungal (*Candida*) in-growth on ~ 1-cm extra-fistula segment, starting from the inside lumen and spreading outwards. Similar bulging was observed in another four A-Tubes, but as the A-Tube needed to be shortened (to accommodate the patient’s weight loss), the problematic section was removed and hence, the A-Tube did not need to be replaced.

### Procedural Success

A total of 202 endoscopies to place the A-Tube were attempted on 201 participants, in aggregate, resulting in 199 successful A-Tube placements. In one participant, the gastrostomy was aborted due to inadequate transillumination. From a subsequent CT scan of the participant’s gastrointestinal tract, the endoscopist noted an unusual orientation of the participant’s stomach, decided to attempt another gastrostomy, was able to achieve adequate transillumination on the second attempt, and proceeded with a successful gastrostomy. In the second and third participants, the gastrostomy was aborted due to discovery of gastric varices in one case, and the liver blocking safe access to the stomach in the other case.

### Discontinuation of Therapy

As of December 31, 2016, 155, 82, 24, and 12 participants completed 1, 2, 3, and 4 years of therapy, respectively. Of the 199 successful gastrostomies, 47 participants discontinued aspiration therapy and had their gastrostomy tubes removed; 17, 18, 9, 2, and 1 participants in the first, second, third, fourth, and fifth year, respectively. Approximately 1/3rd of the 47 “discontinued participants” experienced considerably less weight loss (%TWL) than the mean weight loss of their peers, while 2/3rd of such participants experienced approximately the same or greater weight loss than the mean weight loss of their peers (Suppl. Figure S4). Reasons cited by participants for discontinuation of the therapy include (i) achievement of goal weight (*n* = 13), (ii) inability or unwillingness to adhere to therapy (*n* = 9), (iii) fatigue with the therapy (*n* = 7), (iv) discomfort with the device (*n* = 7), (v) the decision to pursue bariatric surgery (*n* = 5), (vi) unrelated health issues (*n* = 3), (vii) personal economic issues (*n* = 1), (viii) incompatibility with work schedule (*n* = 1), and (ix) unknown (*n* = 1). It should be noted that the five participants who chose to undergo bariatric surgery did so successfully. Applying a Kaplan-Meier “survival” analysis (Suppl. Figure S5) to the withdrawal data, 91, 76, 64, and 53% of the participants who started aspiration therapy persisted with the therapy for 1, 2, 3, and 4 years, respectively.

## Discussion

The mean total weight loss, excess weight loss, and absolute weight loss for participants who completed 1-year of aspiration therapy (AT) was 18.2%, 46.3%, and 22.7 kg, respectively, and was comparable to the weight loss reported in prior AT studies. The mean weight loss for participants who completed a second, third, and fourth year of AT was greater or equal to the first-year weight loss, implying excellent durability in weight loss for at least 4 years. Participants with higher baseline BMIs (> 50) and older participants (> 55 years) showed equal weight loss (%TWL) to their lower BMI and younger counterparts, respectively. The weight loss and durability findings in this study are significant because the study was conducted in a community setting, in which adjunctive lifestyle counseling and visit frequency were typical for “real-world” participants outside of a clinical trial setting.

Participants in this study, after 3–4 months of AT, consistently reported a significant decrease in portion sizes due to the requirement to chew their food thoroughly and therefore eat more mindfully. Although these reports are anecdotal, they are consistent with findings from prior studies [13–15]. Based on bomb calorimetry studies, Sullivan et al. found that aspiration would account for only about 80% of the weight loss observed, even if patients were aspirated all three daily meals and did not snack [13]. In practice, given that most patients skip aspirations and snack occasionally, aspiration would account for significantly less than 80% of the observed weight loss and the remainder of the weight loss would be attributed to decreased caloric intake.

At 1 year, clinically and statistically significant reductions in glycated hemoglobin, triglycerides, blood pressure, and fasting blood glucose were observed; total cholesterol, however, increased, for reasons unknown over the 1-year period. For participants with diabetes, HbA1C decreased by 1.0% (*p* = 0.001) from 7.8% at baseline to 6.8% at 1 year.

This study confirmed the findings of prior aspiration therapy studies; that is, the device is remarkably safe, with generally minor side effects, in comparison with other bariatric procedures. Complications, in the periprocedural period, resulted, on a per-participant basis, in 0.045 hospitalizations, no endoscopies, and no surgeries. Complications, in the post-procedural period, resulted, on a per-patient year basis, in no hospitalizations, no surgeries, and only 0.06 endoscopies. The medical interventions required to address the more serious complications associated with aspiration therapy were typically minimally impactful on the participant (a day or so of hospitalization or a minor endoscopy procedure) and minimally costly to the healthcare system. It is also noteworthy that the complications in the post-procedural period rarely interfered with a participant’s daily activities. Consistent with prior AT studies, there was no indication of any degradation of eating behaviors, including compensatory eating, nor any clinically significant metabolic or nutritional deficiency, as measured by blood tests or as may be inferred from participant complaints such as hair loss or fatigue.

Apart from the A-Tube rotation (the internal end rotating from the fundus towards the pylorus), all other complications reported in this study are similar (or less) in type, severity, and frequency as those reported in the literature with PEG tubes [22]. Whereas the literature on persistent fistulas in PEG tubes (primarily in pediatric patients) reports an onset of 8 months post-gastrostomy, in this study, the onset was at 24+ months post-gastrostomy. We have no explanation for the apparent delay in onset of persistent fistulas in AspireAssist participants versus the onset in PEG tube patients. Given the similarity between the AspireAssist A-Tubes and PEG tubes in their construction, placement, and complications (as reported in this study and prior studies), it is highly unlikely that, as the number of aspiration therapy patients or duration of use grows, mechanical or procedural complications different or more severe than those associated with PEG tubes will emerge with this therapy. However, the higher rate of persistent fistulas with time suggests that more aggressive techniques for closure be considered at the time of tube removal for long-duration gastrostomies (> 2 years).

Procedural success in this study was in excess of 99%, consistent with the findings in the PATHWAY Study. Transillumination was rarely problematic, consistent with the reports in the literature of placement of PEG tubes in people with obesity [23, 24].

A striking differentiator between aspiration therapy and bariatric surgery is that although aspiration therapy can be a long-term therapy, patients can elect to terminate aspiration therapy, and later pursue bariatric surgery, or repeat aspiration therapy. Five participants in this study elected after 3 to 14 months of aspiration therapy to undergo gastric bypass; all five participants had successful surgeries. Given that all weight loss procedures have a not-insignificant fraction of patients who are low responders, experience recidivism, or experience unpleasant intractable side effects, reversible procedures may be appealing to patients.

Interestingly, a large percentage of participants persist with aspiration therapy, for at least for several years, even after they achieve their goal weight. The Kaplan-Meier analysis suggests that slightly more than 50% of patients will persist with AT for at least the 4 years. Based upon anecdotal reports from patients as well as connector usage data, patients aspirate 2–3 times per day on average during the first 6 to 9 months of the therapy, 1–2 times per day by the 18th month, and even less in the third and fourth years. We believe that although many patients may not regain weight after discontinuing therapy, it is unrealistic to expect all patients to be able to discontinue therapy and not regain weight. Many patients, however, appear quite content to have AT as a chronic therapy, albeit with only a fraction of their meals aspirated. Our recommendation to patients who have reached their weight loss goal and are considering A-Tube removal is that they cease aspirating for a period of 6 months prior to A-Tube removal and confirm the absence of weight regain prior to A-Tube removal.

Aspiration therapy is a paradigm shift in the treatment of obesity. Whereas bariatric surgery often provides significant “negative feedback” (in the form of nausea, vomiting, diarrhea, dumping, or the sensation of food getting “caught”) if a patient “over-eats” or eats the “wrong foods”, there is no such punitive aspect with aspiration therapy. An AT patient can occasionally enjoy a special meal (such as on holidays) without experiencing the negative symptoms a patient with bariatric surgery would likely experience. The ability to maintain some dietary and lifestyle flexibility and simultaneously maintain control of one’s weight can be very empowering to many patients.

Another differentiator of AT from bariatric surgery is that the patient essentially determines his or her own weight loss trajectory, and hence, health status. Success with AT requires active participation by the patient (e.g., the patient must chew thoroughly and aspirate regularly after meals). Although not all patients with class II or class III obesity are prepared to make the commitment to be successful with aspiration therapy, many benefits accrue to patients and the overall healthcare system when patients assume responsibility for their health. A consistent pattern the investigators of this study have observed is that when an AT patient fails to meet a weight loss goal at a follow-up visit, the AspireAssist patient assumes responsibility for the lack of weight loss; with other weight loss procedures, patients often blame the procedure for lack of weight loss. Medicine today is trending towards patients becoming more active participants in the management of their healthcare [25].

A third differentiator of aspiration therapy from bariatric surgery is that patients can, and occasionally do, temporarily stop using the device, perhaps regaining some of their weight back during this period, and then relatively easily go back on the program of regular aspirations and lose any weight regained during their “an aspiration holiday.” Management of any chronic disease can be tiring to patients, and the ability to “take a holiday” for a few weeks from management of the disease is often a welcome relief to patients. In general, there is little risk in an aspiration therapy patient taking “an aspiration holiday” as long as weight regain does not cause the internal bumper of the A-Tube to press against the gastric wall, which can lead to a buried bumper.

This report has several limitations. The first limitation is that this report has the absence of a control group to provide a comparative base. A second limitation is that only two sites reported cardiometabolic data; however, the weight loss from these two sites was no greater than that of the sites not reporting cardiometabolic data, suggesting that it is unlikely that the results would differ substantially had there been data from all five clinics. Another limitation is that this report only provides results through 4 years of therapy and the number of participants in years 2–4 is less than in year 1; however, the durability of weight loss and relatively narrow band of 95% confidence intervals suggest robustness of the data. With regard to safety, the excellent and consistent safety data between this report and the PATHWAY trial, coupled with the 30 years of widespread usage of the AspireAssist’s nearest analogue, the PEG tube, suggests that longer-term safety results are not apt to substantially differ from the post-procedural results reported here.

## Conclusions

The AspireAssist System is a safe, effective, and durable weight management therapy for persons with class II and class III obesity. This study demonstrates that aspiration therapy is as effective and safe in a community setting as in a research setting. This therapy provides a viable alternative therapy to bariatric surgery for patients who cannot have or refuse to have bariatric surgery. Compared to other endoluminal bariatric therapies, it is a long-term therapy and is applicable to patients with higher BMIs (> 40 kg/m^2^). Although the therapy requires a substantial commitment on the part of the patient, the data suggests that a very large percentage of patients are willing and able to make the commitment to succeed with this therapy. Finally, the ability to perform the gastrostomy on an outpatient basis and the very low incidence of costly, serious complications suggest that aspiration therapy may be a lower cost alternative to bariatric surgery.

## Electronic supplementary material

ESM 1 (DOCX 33 kb)

Figure S1 Percent total weight loss (%TWL) at 1-4 years for 1-year, 2-year, 3-year, and 4-year completers. (GIF 35 kb)

High resolution image (EPS 519 kb)

Figure S2 **Kaplan Meier survival curve of A-Tubes in situ versus time.** Note that at 48-months in situ, approximately 2/3rds of the A-Tubes are still patent. **(GIF 15 kb)**

High resolution image (EPS 485 kb)

Figure S3a A-Tube with fungal-ingrowth while in situ (JPEG 377 kb)

Figure S3b Cut-away of A-Tube with fungal-ingrowth (GIF 174 kb)

High resolution image (TIFF 4295 kb)

Figure S4 Percent total weight loss (%TWL) at the time of withdrawal for each discontinued participant vs. mean %TWL of the per protocol population (%TWL) (GIF 15 kb)

High resolution image (EPS 514 kb)

Fig. S5 Percent of participants continuing Aspiration Therapy with time using a Kaplan Meier survival analysis. (GIF 15 kb)

High resolution image (EPS 493 kb)
