# Supplementary material for: Aspiration Therapy As a Tool to Treat Obesity: 1- to 4-Year Results in a 201-Patient Multi-Center Post-Market European Registry Study
Source: Obes Surg. 2018 Feb 1;28(7):1860–8. doi: 10.1007/s11695-017-3096-5 (PMC6018576; doi:10.1007/s11695-017-3096-5)
Supplement: Supplementary file 1 — (DOCX 33 kb) [file 11695_2017_3096_MOESM1_ESM.docx]

**Supplementary Appendix**

**Tables:**

**Table S-1. Protocol deviations**

**Table S-2. Percent total weight loss (%TWL) at 1-4 years for participants > 55 years old versus participants < 55 years old.**

**Table S-3. Percent total weight loss (%TWL) at 1-4 years for participants with body mass index (BMI) > 50 kg/m^2^ old versus participants with BMI < 50 kg/m^2^**

**Table S-4. Number of persistent fistula reports versus time since gastrostomy compared to number of A-Tube removals**

**Figures:**

**Fig. S-1. Percent total weight loss (%TWL) at year 1-4 for 1-year, 2-year, 3-year, and 4-year completers**

**Fig. S-2. Kaplan Meier survival curve of A-Tubes in situ versus time.**

**Fig. S-3a. A-Tube with fungal in-growth while in-situ**

**Fig. S-3a. Cut-away of A-Tube with fungal in-growth**

**Fig. S-4. Percent total weight loss (%TWL) at the time of withdrawal for each discontinued participant vs. mean %TWL of the per protocol population (%TWL)**

**Fig. S-5. Percent of participants continuing Aspiration Therapy with time using a Kaplan Meier survival analysis.**

**Table S-1. Protocol deviations/ variations**

| **Type** | **Description** | **Impact** |
| --- | --- | --- |
| **VLCD Diet** | In conformance with the subject clinic’s bariatric protocol, ~75 participants were given a very low calorie diet (800 calories) (VLCD) for 4 weeks prior to original A-Tube placement to shrink the liver. | Although participants on average lost 5-10% TBL on this diet, the literature shows that a VLCD diet has minimal or no impact on long-term weight loss |
| **Intubation/ Overnight** | 2 participants with BMIs above 60 were intubated, and 7 participants with BMIs over 60 were kept overnight for observation | It is not clear if intubation or an overnight stay reduces complications for this population; however, it’s best to err on the side of caution with this population. |
| **Liquid/ Soft Diet** | Approximately 50 participants were placed on a diet of pureed foods the 1^st^ week and a finely chopped diet the 2^nd^ week from beginning AT. The rationale for this is to allow participants to (i) get a sense of what a good aspiration looks like and (ii) learn to master the apparatus without having to master thorough chewing | This initial diet appears to make the learning curve shorter for patients. The labelling for the AspireAssist now includes this diet. [12] |
| **Placement of Skin-Port the day after A-Tube placement** | Approximately 50 participants had their Skin-Ports placed the day after A-Tube Placement, as opposed to 10-14 days. The belief was that placement of the Skin-Port would enhance participant comfort. | It is not clear if early placement was beneficial. A potential concern of early placement is increased risk of a buried bumper due to post-procedure swelling. |

**Table S-2. Percent total weight loss (%TWL) at 1-4 years for participants > 55 years old versus participants < 55 years old.**

|  | **Age >55 years**  **Mean Age** | | **Age <55 years**  **Mean Age** | | **Difference in %TWL**  **(p-value)** |
| --- | --- | --- | --- | --- | --- |
|  | **N** | **%TBL** | **N** | **%TBL** |  |
| **Yr 1** | **35** | **18.2%+8.7%** | **120** | **18.2%+11.0%** | **0.1% (p=1.0)** |
| **Yr 2** | **15** | **21.2%+9.8%** | **67** | **19.5%+13.2%** | **1.7% (p=0.7)** |
| **Yr 3** | **5** | **25.9%+6.2%** | **19** | **20.1%+11.8%** | **5.8% (p=0.3)** |
| **Yr 4** | **2** | **28.2%+9.7%** | **10** | **17.4%+8.7%** | **10.8% (p=0.2)** |

|  |  |  |  |  |  |  |  |
| --- | --- | --- | --- | --- | --- | --- | --- |
|  |  |  |  |  |  |  |  |
|  |  |  |  |  |  |  |  |
|  |  |  |  |  |  |  |  |
|  |  |  |  |  |  |  |  |
|  |  |  |  |  |  |  |  |
|  |  |  |  |  |  |  |  |
|  |  |  |  |  |  |  |  |
|  |  |  |  |  |  |  |  |
|  |  |  |  |  |  |  |  |
|  |  |  |  |  |  |  |  |
|  |  |  |  |  |  |  |  |
|  |  |  |  |  |  |  |  |
|  |  |  |  |  |  |  |  |
|  |  |  |  |  |  |  |  |
|  |  |  |  |  |  |  |  |
|  |  |  |  |  |  |  |  |
|  |  |  |  |  |  |  |  |
|  |  |  |  |  |  |  |  |
|  |  |  |  |  |  |  |  |
|  |  |  |  |  |  |  |  |

**Table S-3. Percent total weight loss (%TWL) at 1-4 years for participants with body mass index (BMI) > 50 kg/m^2^ old versus participants with BMI < 50 kg/m^2^.**

|  | **BMI >50 kg/m^2^**  **Mean BMI** | | **BMI <50 kg/m^2^**  **Mean BMI** | | **Difference in %TWL**  **(p-value)** |
| --- | --- | --- | --- | --- | --- |
|  | **N** | **%TBL** | **N** | **%TBL** |  |
| **Yr 1** | **22** | **18.7%+9.5%** | **133** | **18.1%+10.8%** | **0.6% (p=0.8)** |
| **Yr 2** | **16** | **24.2%+10.7%** | **66** | **18.7%+12.9%** | **5.5% (p=0.2)** |
| **Yr 3** | **7** | **24.8%+10.1%** | **17** | **19.9%+11.8%** | **4.9% (p=0.4)** |
| **Yr 4** | **2** | **25.2%+10.2%** | **10** | **18.0%+8.7%** | **7.2% (p=0.2)** |

**Table S-4. Number of persistent fistula reports versus time since gastrostomy compared to number of A-Tube removals**

| **Time Since Gastrostomy** | **Number of A-Tube Removals** | **Number of Persistent Fistulas** | **Percent Persistent Fistulas to A-Tube Removal** |  |
| --- | --- | --- | --- | --- |
| First Year (0-11.9 Months) | 17 | 0 | 0% |  |
| Second Year (12.0-23.9 Months) | 18 | 1 | 5% |  |
| Third Year (24.0-35.9 Months) | 9 | 2 | 22% |  |
| Fourth Year (36.0-47.9 Months) | 2 | 1 | 50% |  |
| Fifth Year (48.0 -59.9 Months) | 1* |  |  |  |
| **TOTAL** | **47** | **4** |  | |

***No participant has completed five years if therapy as of March 31, 2017**

**Figure S-1. Percent total weight loss (%TWL) at 1-4 years for 1-year, 2-year, 3-year, and 4-year completers.**

**Figure S-2. Kaplan Meier survival curve of A-Tubes in situ versus time.** Note that at 48-months in situ, approximately 2/3rds of the A-Tubes are still patent.

**Figure S-3a. A-Tube with fungal-ingrowth while in situ**

**Figure 3b. Cut-away of A-Tube with fungal-ingrowth**

**Figure S-4. Percent total weight loss (%TWL) at the time of withdrawal for each discontinued participant vs. mean %TWL of the per protocol population (%TWL)**

**Fig. S-5. Percent of participants continuing Aspiration Therapy with time using a Kaplan Meier survival analysis.**
