# Supplementary figures and images for: Aspiration Therapy As a Tool to Treat Obesity: 1- to 4-Year Results in a 201-Patient Multi-Center Post-Market European Registry Study
Source: Obes Surg. 2018 Feb 1;28(7):1860–8. doi: 10.1007/s11695-017-3096-5 (PMC6018576; doi:10.1007/s11695-017-3096-5)

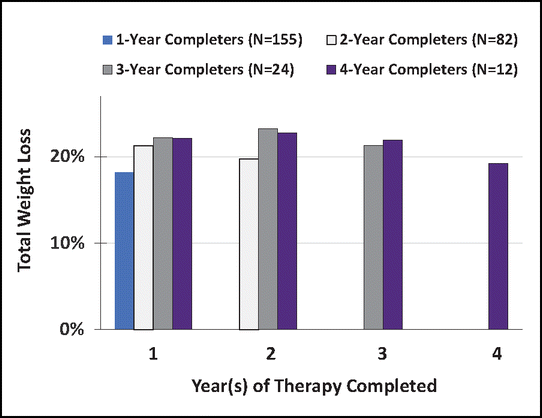

Supplement: Supplementary file 2 — Percent total weight loss (%TWL) at 1-4 years for 1-year, 2-year, 3-year, and 4-year completers. (GIF 35 kb) [file 11695_2017_3096_Fig3_ESM.gif]

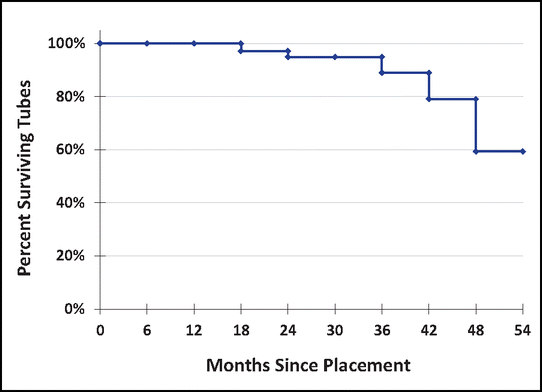

Supplement: Supplementary file 4 — Kaplan Meier survival curve of A-Tubes in situ versus time. Note that at 48-months in situ, approximately 2/3rds of the A-Tubes are still patent. (GIF 15 kb) [file 11695_2017_3096_Fig4_ESM.gif]

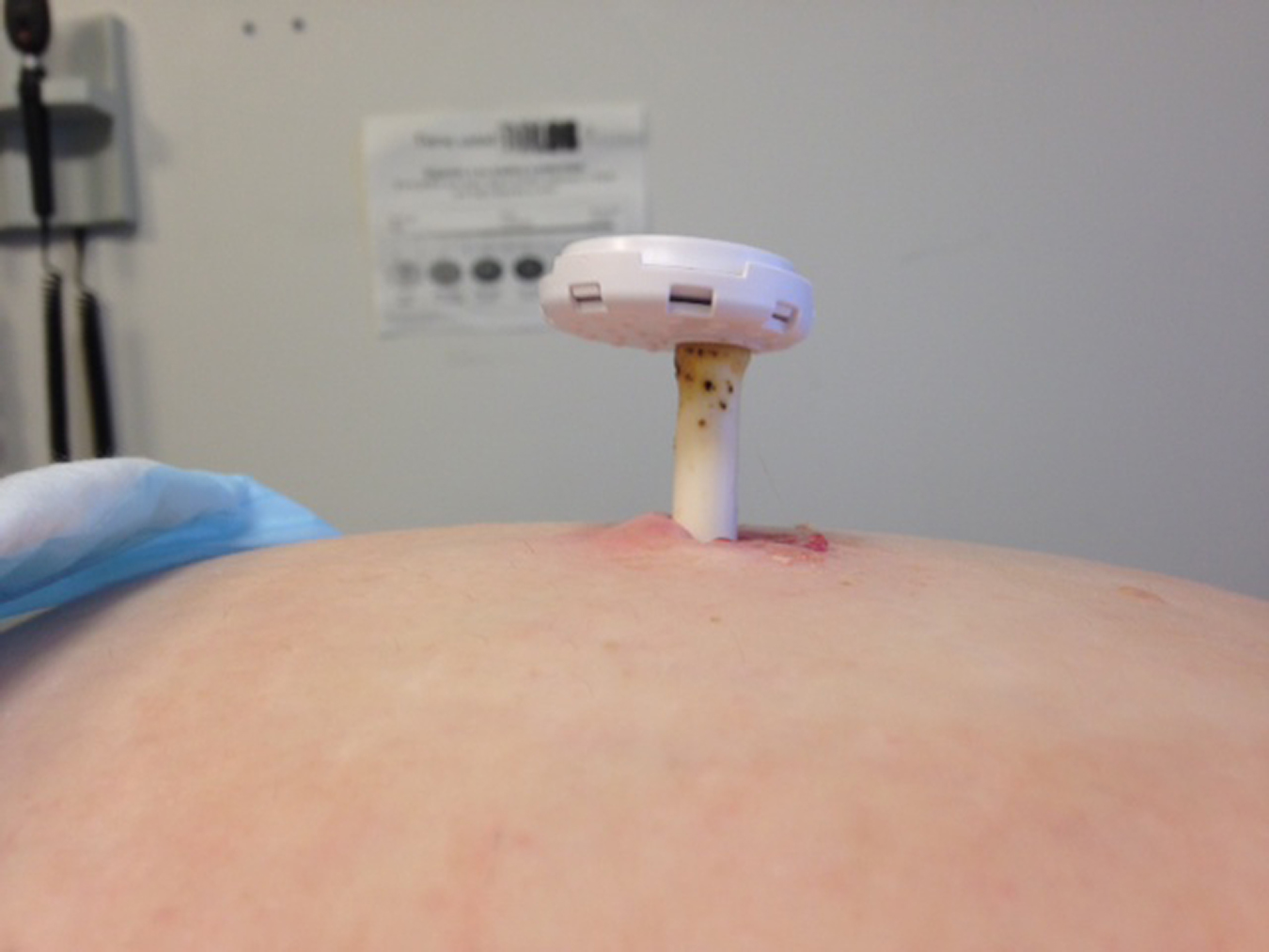

Supplement: Supplementary file 6 — A-Tube with fungal-ingrowth while in situ (JPEG 377 kb) [file 11695_2017_3096_MOESM4_ESM.jpg]

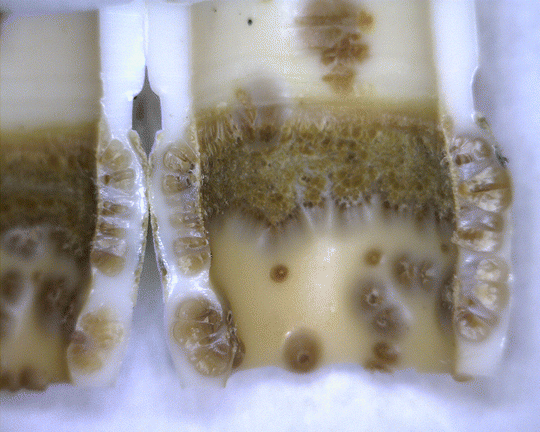

Supplement: Supplementary file 7 — Cut-away of A-Tube with fungal-ingrowth (GIF 174 kb) [file 11695_2017_3096_Fig5_ESM.gif]

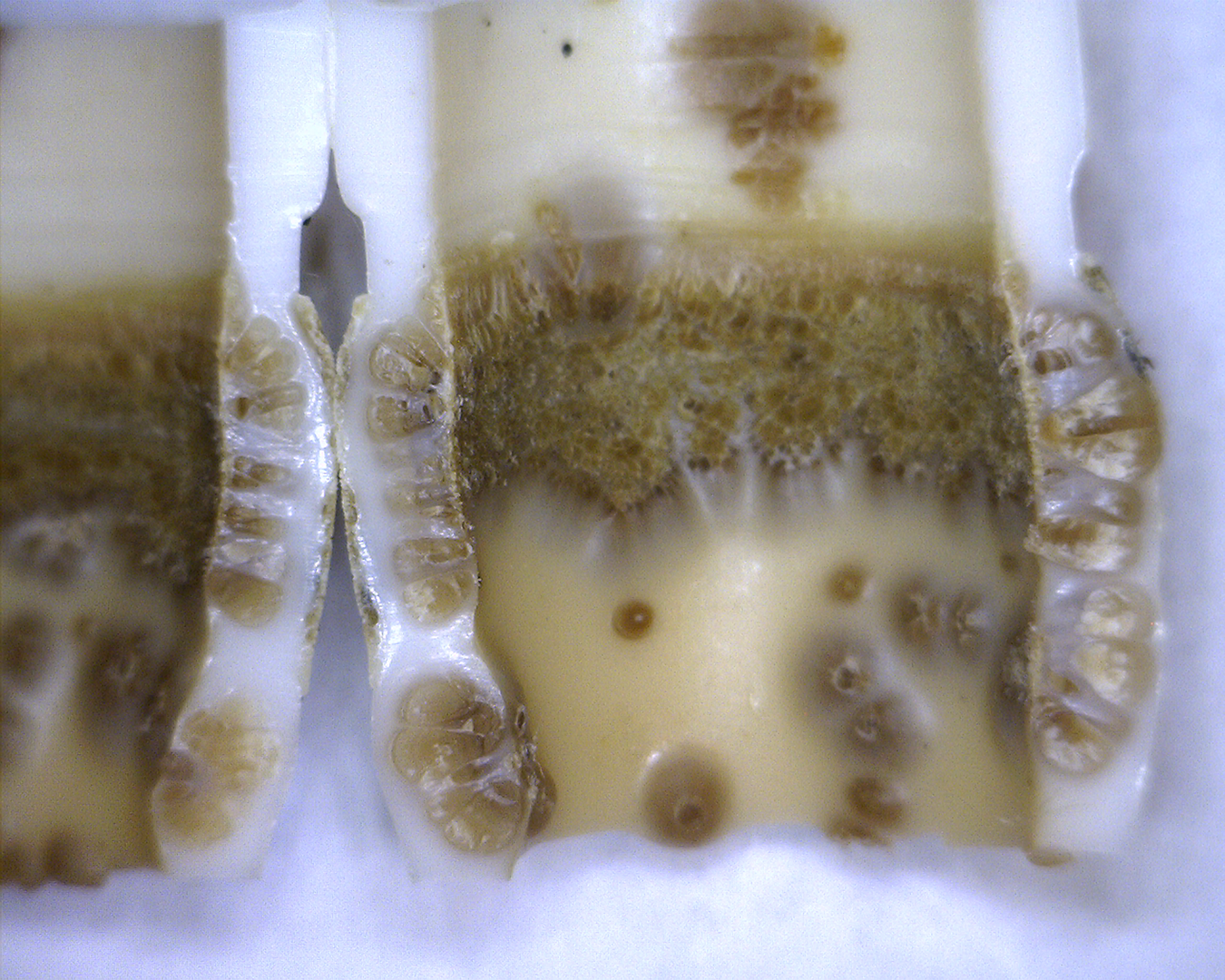

Supplement: Supplementary file 8 — High resolution image (TIFF 4295 kb) [file 11695_2017_3096_MOESM5_ESM.tif]

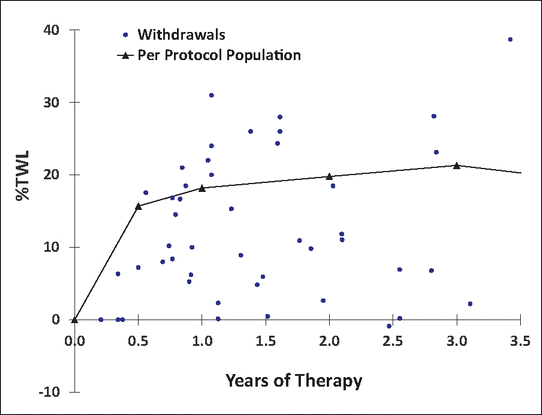

Supplement: Supplementary file 9 — Percent total weight loss (%TWL) at the time of withdrawal for each discontinued participant vs. mean %TWL of the per protocol population (%TWL) (GIF 15 kb) [file 11695_2017_3096_Fig6_ESM.gif]

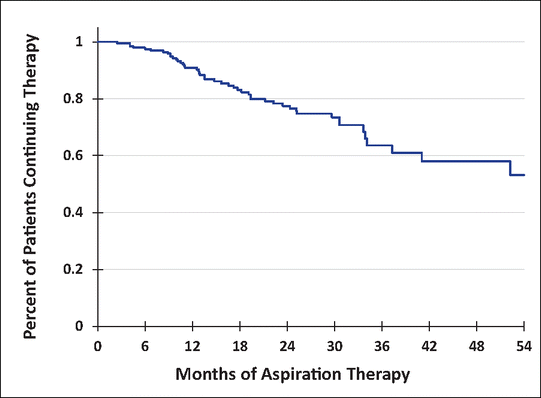

Supplement: Supplementary file 11 — Percent of participants continuing Aspiration Therapy with time using a Kaplan Meier survival analysis. (GIF 15 kb) [file 11695_2017_3096_Fig7_ESM.gif]
